# Rational design of magnetically separable core/shell Fe_3_O_4_/ZnO heterostructures for enhanced visible-light photodegradation performance

**DOI:** 10.1039/d1ra03468e

**Published:** 2021-06-24

**Authors:** Hoai Linh Pham, Van Dang Nguyen, Van Khien Nguyen, Thi Hong Phong Le, Ngoc Bach Ta, Do Chung Pham, Quoc Toan Tran, Van Thanh Dang

**Affiliations:** Institute of Materials Science, Vietnam Academy of Science and Technology Hanoi Vietnam; Graduate University of Science and Technology, Vietnam Academy of Science and Technology Hanoi Vietnam; Department of Physics and Technology, TNU-University of Sciences Thai Nguyen Vietnam dangnv@tnus.edu.vn; Department of Physics, Hanoi National University of Education 136 Xuan Thuy Road, Cau Giay District Hanoi 100000 Vietnam; Department of Chemistry, Thai Nguyen University of Education Vietnam; TNU – University of Medicine and Pharmacy Vietnam thanhdv@tnmc.edu

## Abstract

Magnetically separable core/shell Fe_3_O_4_/ZnO heteronanostructures (MSCSFZ) were synthesized by a facile approach, and their application for enhanced solar photodegradation of RhB was studied. The formation mechanism of MSCSFZ was proposed, in which Fe_3_O_4_ nanoparticles served as a template for supporting and anchoring the ZnO crystal layer as the shells. The morphology of MSCSFZ can be varied from spherical to rice seed-like structures, and the bandgap was able to be narrowed down to 2.78 eV by controlling the core–shell ratios. As a result, the MSCSFZ exhibited excellent visible-light photocatalytic activity for degradation of rhodamine B (RhB) in aqueous solution as compared to the controlled ZnO nanoparticles. Moreover, MSCSFZ could be easily detached from RhB solution and maintained its performance after 4 cycles of usage. This work provides new insights for the design of high-efficient core/shell recyclable photocatalysts with visible light photocatalytic performance.

## Abstract 2

Magnetically separable core/shell Fe_3_O_4_/ZnO heteronanostructures (MSCSFZ) were synthesized by a facile approach, and their application for enhanced solar photodegradation of RhB was studied.

## Graphical abstract

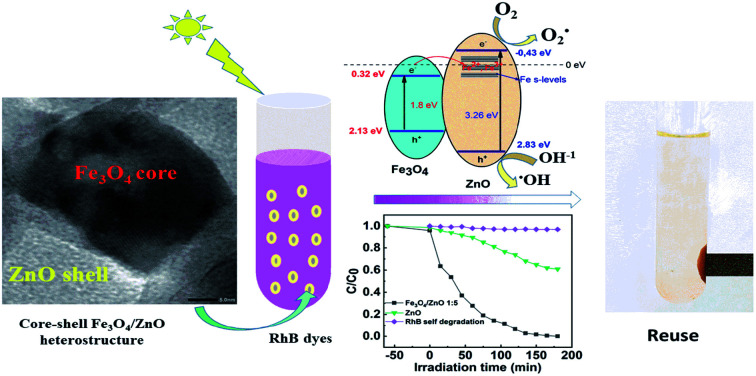

## Introduction

Utilizing nanomaterials for water treatment technology has been considered as a potential solution for protecting and developing the environment and energy resources.^1^ With the rapid development of water treatment nanotechnology, semiconductor nanophotocatalysts have drawn considerable attention owing to their superior efficiency in photocatalytic degradation of stable and toxic organic pollutants in water sources.^2,3^ The photocatalysis mechanism is based on light absorption of semiconductor materials to create electrons and holes in the conduction band and valance band. These photo-inducted charge carriers can move to the surface of semiconductors and act as strong reductants and oxidants to generate reactive oxygen species for degradation of pollutants.^4^ From previous studies, it is evident that photocatalysis possess outstanding advantages compared to other treatment techniques such as (i) utilize solar energy; (ii) suitable operation conditions (temperature and pressure), (iii) high efficiency with completely decomposing the stable and toxic organic substances without generating any secondary pollutants; (iv) low operating cost; (v) save time.^4–6^

Over the years, a wide range of single semiconductors were used for photocatalytic technology.^7^ However, recently, a various type of heterogeneous photocatalysis composed by two or more semiconductors were rapidly developed to overcome draw-backs of single semiconductors as: enhancing visible photocatalytic activity with suitable band edges and improving photo-generated charge carrier separation; easy collecting in the solution.^5,7–9^ An alternative to benchmark TiO_2_, ZnO is also an well-known photocatalyst for degradation of organic pollution in aqueous media because of its higher electron mobility, longer lifetime of charge carriers, and especially in low cost and environmental friendliness.^10,11^ Besides, the structure–morphology of ZnO can be easily varied by wet-chemistry strategy to obtain the well-defined structures for photocatalytic and sensing applications. Although ZnO has been widely served as an efficient and low-cost photocatalyst in water remediation, however, there are some problems needed to be solved, such as (i) the fast photogenerated electron–hole recombination and (ii) the large bandgap energy of 3.37 eV, resulting in low efficiency of solar radiation absorption (from 3% to 5%); subsequently, the photocatalytic activity is unsatisfactory for the applications in industry.^12^

To overcome these limitations, recent efforts have been focused on modifying the electronic band structure of ZnO by doping transition/noble metals or combining ZnO with other materials to form a hybrid photocatalyst system such as low bandgap semiconductors and magnetic nanomaterials.^13–15^ Currently, the synthesis of heterogeneous structures between ZnO and magnetic nanomaterials has gained some interest in the field.^16–18^ The combination of ZnO and several spinel ferrite nanomaterials has created a new hybrid material that is capable to enhance the visible light photocatalytic efficiency and advantageous in recovery and reusability.^19–22^ Typically, nanoscale materials have a primary bottleneck in recovery and reuse since they are in particulate form in solution, which hinders the outstanding performance in practical industrial environment. The nanocomposites of photocatalysts with magnetic-based materials allows detaching the nanocomposites from aqueous treatment solution by simply applying an external magnetic fields. Various metal magnetic oxides, such as FeO, Fe_2_O_3_, Fe_3_O_4_ or ferrites of MFe_2_O_4_ (M = Co, Mn, Ni) are widely used for incorporating with ZnO to form the magnetically recoverable photocatalysis.^23^ Among the magnetic-based materials, Fe_3_O_4_ possesses the highest saturation magnetization (theoretical value of 93 emu g^−1^ at room temperature), superior adsorption of heavy metals, as well as the excellent ability to decompose organic pollutants.^24,25^ The combination of Fe_3_O_4_ and ZnO will formed a heterojuntion in which electrons and holes of two materials can be transfer to others based on the energy band position. These lead to an effective separation of photogenerated charge carrier and, in some cases, extends absorption of visible-region light.^4,5,8^ It is expected that the core/shell structured Fe_3_O_4_/ZnO at the nanoscale not only exhibits good magnetic separability, but also suppresses the charge carrier recombination, and achieving a narrow bandgap of ZnO for reaching visible-light-driven photocatalysis. So far, there has been much literature on fabrication of core/shell nanostructured Fe_3_O_4_/ZnO for photocatalytic applications. However, to the best of our knowledge, the previous works mainly reported on the effective degradation of organic pollutants under ultraviolet irradiation, while the visible light-driven photocatalytic performance of core/shell nanostructure is still in its fancy. Additionally, the ratio between Fe : Zn in hybridization is believed to tune the overall bandgap and thus enhance the photocatalytic performance. Nonetheless, there is a gap knowledge in this field due to the lack of synthesis control over Fe : Zn ratio. Therefore, it is imperative to comprehensively study on the effects of Fe_3_O_4_/ZnO ratio as a core/shell heterostructures for photocatalytic degradation of organic pollutants under the visible light irradiation.

In this work, we reported a facile approach to synthesize core/shell nanostructured Fe_3_O_4_/ZnO magnetic photocatalyst with tunable band gap for photodegradation of rhodamine B (RhB) under sunlight irradiation. The material properties were characterized by using SEM, TEM, UV-Vis, XRD, and Raman. By controlling the Fe : Zn molar ratio, different morphologies of Fe_3_O_4_/ZnO were achieved. The Fe_3_O_4_/ZnO heterostructures with the optimal core/shell Fe : Zn ratio showed excellent visible light photo-degradation activity of RhB. The reusability and recovery have also been demonstrated after four cycles.

## Experimental

### Chemicals

Chemicals including ferrous chloride (FeCl_2_·4H_2_O, 99%), ferric chloride hexahydrate (FeCl_3_·6H_2_O, 99%), zinc acetate dihydrate (Zn(CH_3_COO)_2_·2H_2_O, 98%), and sodium carbonate (Na_2_CO_3_, 99.95%) were purchased from ACROS Organics™ (USA). HCl (36.5–38.0%), ammonium hydroxide solution (NH_4_OH, 28–30%), diethylene glycol (C_4_H_10_O_3_, 99%), ethylene glycol – EG (C_2_H_6_O_2_, 99%), citric acid – CA (C_6_H_8_O_7_, 99%) were supplied by Sigma Aldrich.

### Preparation of Fe_3_O_4_ nanoparticles

Fe_3_O_4_ magnetic nanoparticles (MNPs) were synthesized by co-precipitation. The salts of FeCl_2_·4H_2_O (0.795 g) and FeCl_3_·6H_2_O (2.61 g) with a molar ratio of 1 : 2 were dissolved in a beaker containing 30 mL of EG–CA solution (2.56 g of CA) under vigorous stirring within 1 hour. The beaker was further added by dropwise NH_4_OH solution (3 M) at 90 °C until the reaction was completed (black color). The MNPs were washed with deionized (DI) water by a magnet and re-dispersed in 100 mL of EG–CA solution by sonication.

### Preparation of core/shell Fe_3_O_4_/ZnO heterostructures

In a typical procedure, various amounts of Zn(CH_3_COO)_2_·2H_2_O were dissolved into 100 mL of a solution including 75 mL of distilled water and 25 mL of DEG under mechanical stirring, followed by the addition of 25 mL of EG–CA mixed solution containing the dispersed Fe_3_O_4_ NPs at room temperature. After stirring for 2 h, Na_2_CO_3_ (1.5 M) solution was added dropwise to the above solution, and then continuously stirred for 1 h. Subsequently, the reaction product was separated by centrifugation, washed with copious DI water, and dried in an oven for 24 h at 60 °C. The final product was annealing at 500 °C for 2 h with a ramping rate of 5 °C min^−1^. To investigate the influence of the core–shell ratio of Fe_3_O_4_/ZnO heterostructure, a series of samples with different Fe : Zn molar ratios were prepared as 1 : 5, 1 : 10, and 1 : 20. During synthesis, the amount of Fe_3_O_4_ NPs was fixed, while the amount of ZnO varied by changing the concentration of zinc precursor. The core/shell samples with Fe : Zn molar ratios of 1 : 5; 1 : 10; 1 : 20 were named as Fe_3_O_4_/ZnO 1 : 5; Fe_3_O_4_/ZnO 1 : 10 and Fe_3_O_4_ : ZnO 1 : 20, respectively.

### Characterization

The crystalline structure of core/shell nanostructured Fe_3_O_4_/ZnO was characterized by X-ray diffraction (XRD) by using a Bruker D8 Advance X-ray diffractometer with Cu-Kα radiation (*k* = 1.5406 Å) at 2*θ* range of 10–70°. The X'pert Highscore Plus program was carried out to evaluate the grain size of the MNPs. The morphological characteristics, shape, and size of the MNPs and the core/shell nanostructure were observed through field emission scanning electron microscopy (FESEM; Hitachi S-4800) equipped with an energy dispersive X-ray spectrometer, and high-resolution transmission electron microscope (JEM 2100, JEOL). Fourier transform infrared (FTIR) spectroscopy (FTIR-GBC Cintra 40 Nicolet Nexus 670 FTIR), Raman spectra (XploRA, Horiba) and X-ray photoelectron spectroscopy measurement (Mutilab-2000 spectrometer with an Al Kα monochromatized source) were carried out to investigate the interaction between the Fe_3_O_4_ MNP core and ZnO shell. The optical properties of samples were characterized by UV-vis-NIR absorption spectroscopy (Hitachi U-4100) and HR photoluminescence system (IHR 500, Jobin Yvon) with an excitation wavelength of 350 nm. A commercial VSM (MicroSence EZ9) was used to observe the magnetic properties and saturation magnetization. Hysteresis loops were determined at an applied field of up to ±18 kOe at room temperature.

### Photocatalytic activity experiment

Photocatalytic activity of the as-prepared samples was investigated by the degradation of RhB solution (a test contaminate) under solar light irradiation (AM 1.5G, Newport). Typically, 60 mg of the as-synthesized catalyst was added into 60 mL of RhB solution (10 ppm) under vigorous stirring in the dark for at least 1 h to achieve the adsorption–desorption equilibrium. The mixture was irradiated by solar light, and the distance from the light source to the liquid level of RhB aqueous solution was kept at 15 cm. During irradiation, 2 mL of the reaction mixture was withdrawn at each time interval of 15 min, and photocatalysts were separated from RhB solution by using a magnet. The degradation of RhB solution was monitored by determining the concentration of RhB in solution through absorbance measurements at 554 nm. The degradation efficiency of RhB was calculated using the following equation:1
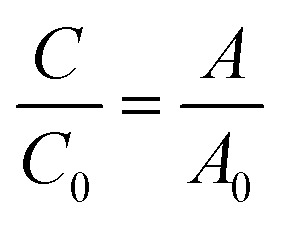
where *C*_0_ and *C* are the initial and real-time concentrations of RhB solution, respectively, and *A*_0_ and *A* are the initial and real-time absorbance of RhB solution, respectively.

## Results and discussion

### Structural and morphological characterizations

**Fig. 1:**
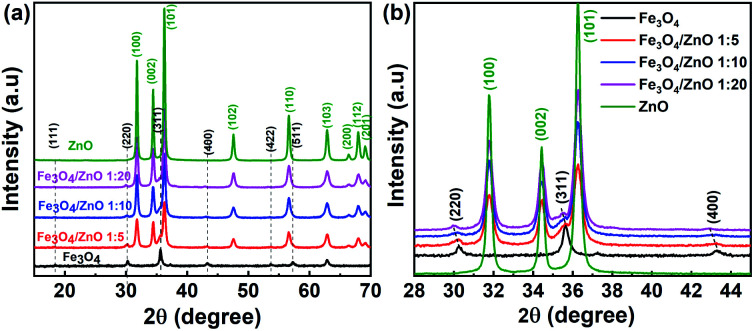
XRD diffraction of (a) the as-prepared samples; (b) the enlarged XRD patterns of samples with 2*θ* angle in the range of 28*–*45°.

The crystal structure and phase purity of the as-prepared samples were identified by XRD. Fig. 1 shows the XRD patterns of the Fe_3_O_4_ NPs, core/shell Fe_3_O_4_/ZnO heterostructures (CSFZ) with different Fe : Zn molar ratios (1 : 5, 1 : 10; and 1 : 20), and ZnO samples. Obviously, the diffraction peaks of Fe_3_O_4_ NPs were observed at 2*θ* of 18.32°, 30.14°, 35,5°, 43.15°, 53.54°, 57.07° corresponding to the (111), (220), (311), (400), (422), (511) planes of the inverse cubic spinel structure of Fe_3_O_4_ crystal, which are well-matched with JCPDS 71-6336. The strong diffraction peaks centered at 2*θ* of 32.77°, 34.44°, 36.26°, 47.55°. 56.6°, 62.88°, 66.38°, 67.96°, 69.09° are indexed to (100), (002), (101), (102), (110), (103), (200), (112), (201) planes of the hexagonal wurtzite structure of ZnO crystalline (JCPDS, 71-6424). For the CSFZ, the diffraction peaks were mainly attributed to the hexagonal wurtzite structure of the ZnO phase, while the small and broad peaks observed at 2*θ* of 30.1°, 35.6°, and 43.2°, corresponding to the (220), (311), and (400) planes of the inverse cubic spinel structure of Fe_3_O_4_, respectively. The appearance of the characteristic peaks of both Fe_3_O_4_ and ZnO crystalline in the XRD patterns of CSFZ samples confirmed for the successful formation of Fe_3_O_4_ and ZnO heterostructure. A closer observation of the XRD patterns in the 2*θ* range of 28°–46° showed that the characteristic peaks of Fe_3_O_4_ phase in the heterostructure samples shifted toward smaller 2*θ* angles as compared with that of the original one (Fig. 1b), while the diffraction peaks of the ZnO shell were still unchanged. According to Bragg's law, the shift in diffraction peaks to lower 2*θ* can be related to the increase in lattice strain, implying the significant expansion of the Fe_3_O_4_ crystal distance in the heterostructure samples. This is due to the hybridization of core/shell at the interface as a result of the formation of core/shell structure that ZnO shell was grown on the surface of Fe_3_O_4_ core.^26^ The average crystallite size was performed by X'pert Highscore Plus and shown in Table 1. The calculated results are in good agreement with the above mentioned findings.

**Table 1:** Microstructural parameters of the as-prepared samples: the lattice spacing of (220) plane of Fe_3_O_4_ crystal (*d*_220_ (Fe_3_O_4_)), average crystallite size (*D*_XRD_) and particle size estimated from FESEM images (*D*_FESEM_)

Samples	*d*_220_ (Fe_3_O_4_)	*D*_XRD_ (nm)		Morphology	*D*_FESEM_ (nm)
		Fe_3_O_4_ core	ZnO shell		
Fe_3_O_4_	2,95 164	12.8	—	Spherical	15.8
Fe_3_O_4_/ZnO 1 : 5	2,96 648	10.86	19.8	Nearly spherical	39
Fe_3_O_4_/ZnO 1 : 10	2,97 249	10.34	20.1	Rice seed-like	*W*: 42; *L*: 96
Fe_3_O_4_/ZnO 1 : 20	2,97 513	11.6	22.03	Rice seed-like, rods	*W*: 40; *L*: 139
ZnO	—	36.49		Nearly spherical	87

**Fig. 2:**
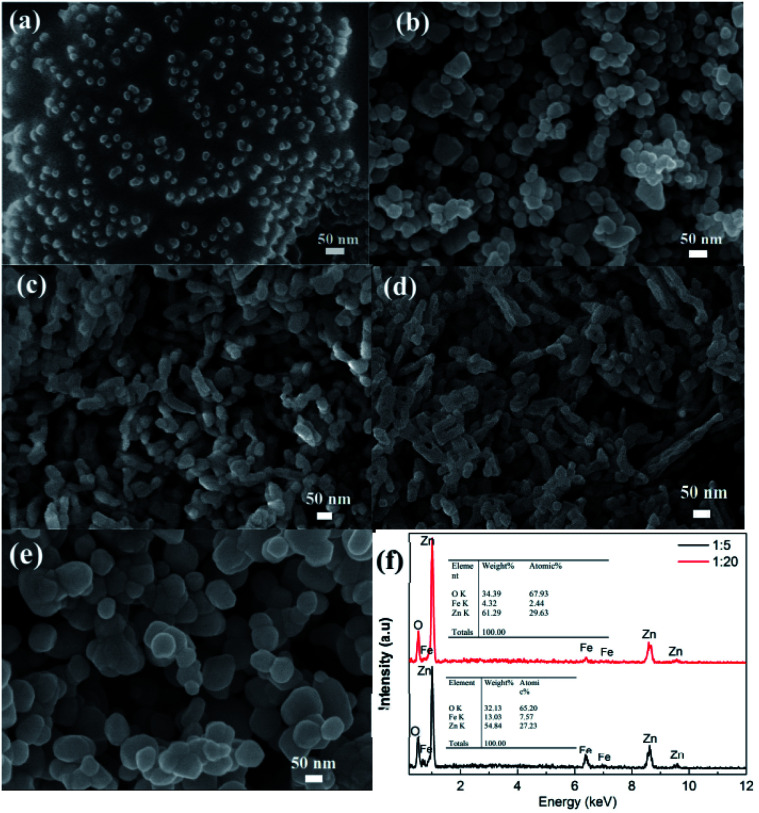
FESEM images of (a) uncoated Fe_3_O_4_ dispersed in EG–CA solution; (b) heteronanostructure samples with different Fe : Zn molar ratio: (b) Fe_3_O_4_/ZnO 1 : 5, (c) Fe_3_O_4_/ZnO 1 : 10, (d) Fe_3_O_4_/ZnO 1 : 20 and (e) control ZnO; (f) The EDS spectrums of Fe_3_O_4_/ZnO 1 : 5 and Fe_3_O_4_/ZnO 1 : 20.

The FE-SEM was used to investigate the morphological features of all samples. As shown in Fig. 2a, the pristine Fe_3_O_4_ NPs display a uniform spherical shape with an average size of 15.8 nm, thus making them well-dispersed in the EG–CA solution. After being covered by the ZnO shell, the morphology and size of CSFZ (Fig. 2b–d) changed drastically compared to the pristine Fe_3_O_4_ NPs. At Fe/Zn ratio of 1 : 5, the core/shell heterostructure retained nearly spherical morphology with a wide size distribution, and the average particle size was estimated to be 39.8 nm. As increasing the Fe : Zn ratios up to 1 : 10 and 1 : 20, the morphology changes from spherical shape of core/shell structure to rice seed-like structure (Fig. 2c and d). The rice seed-like shaped nanostructures with an average size of 42 nm in diameter and 6 nm in lengths were observed for the Fe_3_O_4_/ZnO 1 : 10 sample. For the Fe_3_O_4_/ZnO 1 : 20 samples, the formation of the nano rod-like structure of CSFZ was seen with average lengths of 139 nm. It should be noted that all samples were synthesized in the same experimental conditions with the same amount of Fe_3_O_4_ core, meanwhile the ZnO shell content varied by adjusting zinc precursor concentration. Thus, the structural evolution of the CSFZ is assigned for the formation of ZnO shell, suggesting the Zn content is crucial for designing the desirable structure. As comparison for ZnO NPs, they displayed a spherical shape with an average diameter size of 87 nm, which is larger than Fe_3_O_4_ NPs (Fig. 2e). To further analyze the elemental composition, EDS analysis was carried out on the Fe_3_O_4_/ZnO 1 : 5 and Fe_3_O_4_/ZnO 1 : 20 samples (Fig. 2f). The results revealed that the core/shell nanostructured Fe_3_O_4_/ZnO included O, Fe, and Zn elements, in which the atomic ratio of Zn to Fe in the EDS spectrum of Fe_3_O_4_/ZnO 1 : 20 sample was higher than that of Fe_3_O_4_/ZnO 1 : 5 sample. It confirmed that the Fe_3_O_4_/ZnO heterostructures with controlled core–shell ratio was successfully synthesized by the proposed method.

**Fig. 3:**
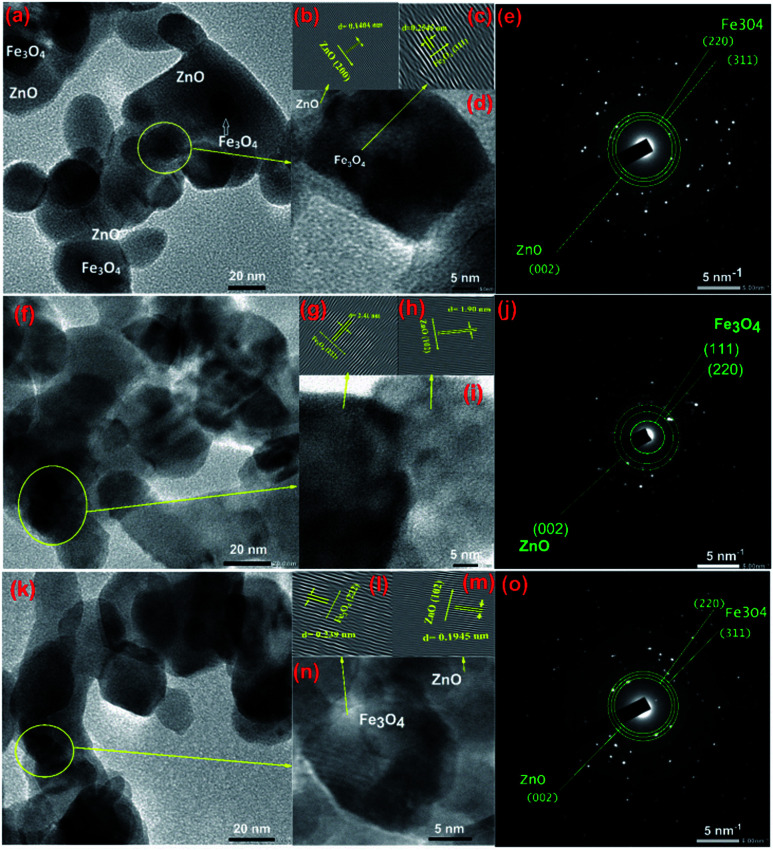
TEM images and HTREM images of Fe_3_O_4_/ZnO 1 : 5 (a and d), Fe_3_O_4_/ZnO 1 : 10 (f and i), Fe_3_O_4_/ZnO 1 : 20 (k and n); the lattice images transferred by inverse fast Fourier transform of Fe_3_O_4_ and ZnO crystals (b and c) for Fe_3_O_4_/ZnO 1 : 5, (g and h) for Fe_3_O_4_/ZnO 1 : 10, (l and m) for Fe_3_O_4_/ZnO 1 : 20; and SAED pattern of Fe_3_O_4_/ZnO 1 : 5 (e), Fe_3_O_4_/ZnO 1 : 10 (j) and Fe_3_O_4_/ZnO 1 : 20 (o).

To gain further insight into the evolution of CSFZ, HR-TEM images of samples with Fe : Zn molar ratio of 1 : 5, 1 : 10 and 1 : 20 were shown in Fig. 3. TEM images shows the features of the core–shell structure formation, in which Fe_3_O_4_ core is dark and ZnO shell is light (different brightness). From HR-TEM images, different lattice fringes were observed in the center and the edge region of a particle, suggesting the existence of a crystalline core/shell structure. The lattice spacing of the core and shell correspond to (311) and (222) plane of Fe_3_O_4_ crystal, and (200) and (102) of ZnO crystal. In addition, the selected area electron diffraction (SAED) images presented the ring patterns of the polycrystalline sample with the lattice spacing of 4.81, 2.98 and 2.53 Å, corresponding to the Fe_3_O_4_ (111), (220), Fe_3_O_4_ (311) reflections, respectively. The lattice spacing of ZnO (002) is also expected to 2.61 Å. These results suggested that the core/shell Fe_3_O_4_/ZnO were well formed in spherical shape at a low Fe : Zn ratio, meanwhile, the ZnO peculates tended to link together to form rice-like or nanorod structures at relatively high Fe : Zn ratios. The mechanism for the structural formation of core/shell Fe_3_O_4_/ZnO heterostructure is proposed as follows: during reaction at second step of sol–gel process, Zn^2+^ ions absorbed on the surface of Fe_3_O_4_ nanoparticles under the linking of surface connecting agents (DEG and EG–CA) and growth as ZnO crystal shell to form the Fe_3_O_4_/ZnO core–shell particles. When increasing the ZnO shell ratio, the more amount of Zn^2+^ ions continue growth on surface of Fe_3_O_4_ NPs and tended to link together to form nanorod structures.

**Fig. 4:**
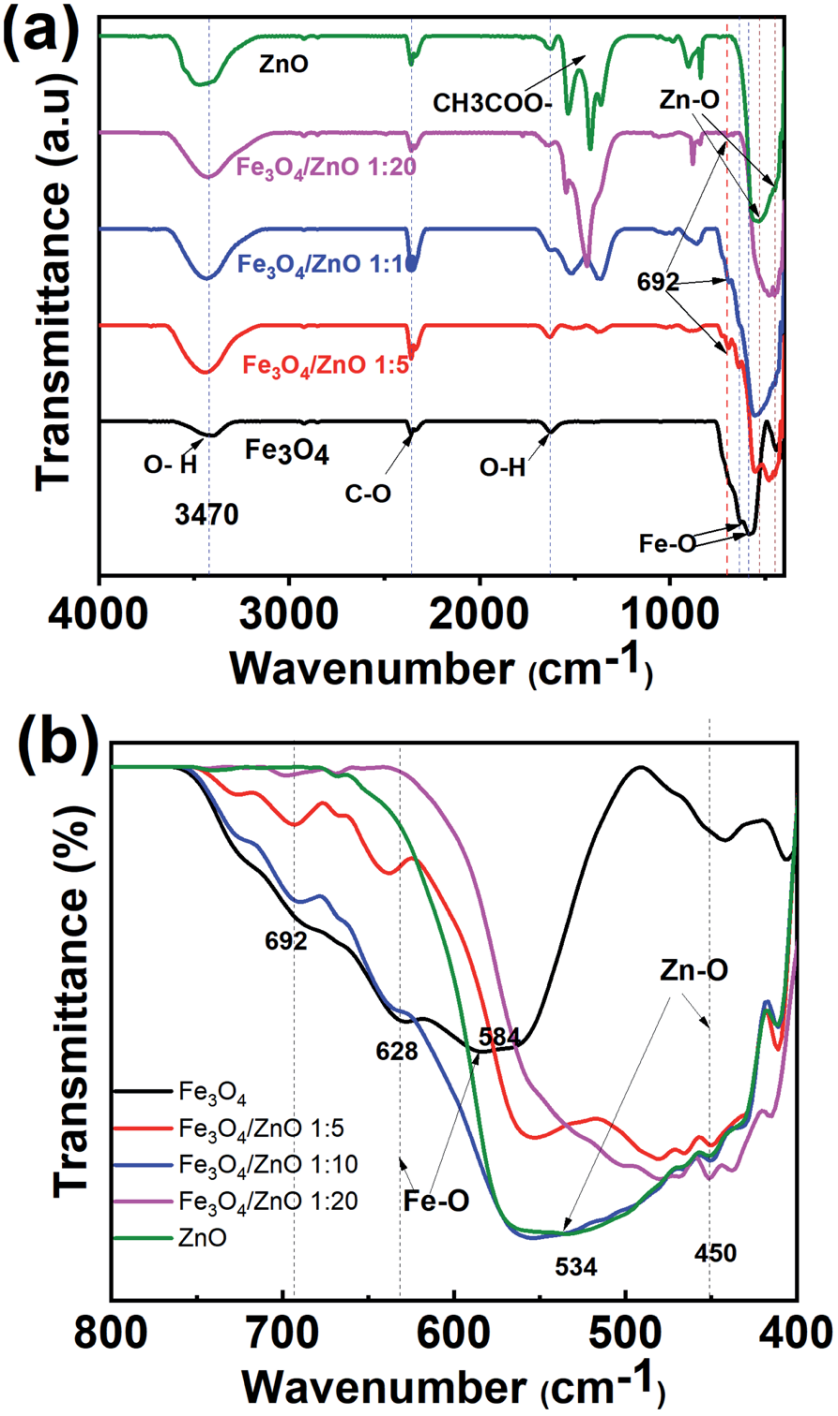
(a) FTIR spectrums of as-prepared samples and (b) the enlarged spectra with wavenumber in the range of 800 cm^−1^ to 400 cm^−1^.

**Fig. 5:**
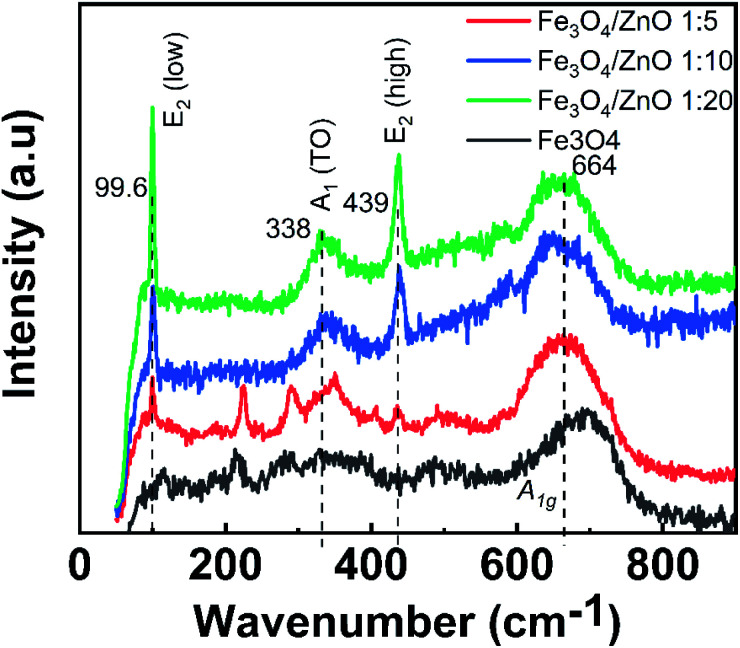
Raman spectra of Fe_3_O_4_ nanoparticles and Fe_3_O_4_/ZnO heterostructures with different Fe : Zn molar ratio.

To gain in-depth understanding about the chemical bonding and vibration, the FTIR analysis of prepared samples was carried out. Fig. 4 presents the FTIR spectra of Fe_3_O_4_ NPs, ZnO NPs and CSFZ with different Fe : Zn molar ratio of 1 : 5, 1 : 10, and 1 : 20. The stretching vibration modes on the FTIR spectra were generally categorized into three wavenumber regions. (i) The stretching vibration modes observed in the wavenumber region from 400 to 900 cm^−1^ correspond to metal–oxygen bonds, which could confirm metal oxide formation. For the pristine Fe_3_O_4_ and ZnO NPs, the bands located at 628–584 and 534–450 cm^−1^ were ascribed to Fe–O bond and Zn–O bond, respectively.^27,28^ Next, the region with wavenumber from 1000 to 1700 cm^−1^, the absorption band centered at 1625 cm^−1^ was attributed to the bending vibration of O–H bond, which is belongs to H_2_O molecules presented in samples. Moreover, the stretching vibrations of the intermediates or residual products, for instance, CH_3_COO… can be found at 1524, 1419, and 1362 cm^−1^.^20,29^ Last, the absorption bands obtained in the region from 2000 to 3700 cm^−1^ were related to the O–H stretching vibration (3385 cm^−1^) and C–O stretching modes (2358–2337 cm^−1^) due to the existence of undesired CO_2_ in the sample during measurements.^30^ Focusing on the low wavenumber region, as shown in Fig. 4b, the absorption peaks assigned to Fe–O bonds and Zn–O bonds in core/shell samples shifted to lower wavenumbers compared with those of pure Fe_3_O_4_ and ZnO NPs. The peak shift revealed that the successful introduction of ZnO NPs on surface of Fe_3_O_4_ NPs to form a core/shell structure, thus requiring the higher energy and resulting in the change of bond length and energy. In addition, a new absorption mode was found at 692 cm^−1^ in core/shell samples, which is associated to the incorporation Fe into the Zn–O lattice due to the hybridization of Fe_3_O_4_ core and ZnO shell at the core–shell interface and similar to previous scholarly publications.^31–33^ In terms of peak intensity located at 692 cm^−1^, the core/shell Fe_3_O_4_/ZnO 1 : 5 exhibited the strongest and sharpest among these compared samples, which further confirms the better core/shell formation at a low precursor Fe : Zn ratio. To verify this unique feature, we employed Raman spectra and their results are shown in Fig. 5. The peaks observed at 99.6, 338 and 439 cm^−1^ were assigned to the E_2_ (low) mode, A_1_ (TO) mode E_2_ (high) mode of ZnO NPs, which is consistent with the previous study.^34^ The broad peak centered at 664 cm^−1^ was attributed to A_1g_ mode corresponding to the symmetric stretch of oxygen atoms along Fe–O bonds in Fe_3_O_4_ NPs. It is well-known that E_2_ (high) and E_2_ (low) modes in ZnO NPs corresponded to the vibrations of oxygen atoms and Zn sub-lattice, respectively.^35^ Evidence by the previous work of Morozov *et al.*, the alter in Raman peak intensity of E_2_ (high) and E_2_ (low) related to the defects on the ZnO side of the interface.^35^ Karamat *et al.* revealed that the intensity of the E_2_ (low) mode decreased due to phonon vibrations of the existence of Fe dopant.^36^ Thus, the decreasing of the intensity of E_2_ (high) and E_2_ (low) mode observed in Raman spectra of core/shell heteronanostructures confirm again the hybridization of Fe_3_O_4_ core and ZnO shell with the Fe ions incorporated in the ZnO crystal lattice at the interface of core–shell. Taken the above discussion, it is strongly confirmed that core/shell nanostructured Fe_3_O_4_/ZnO was successfully prepared.

**Fig. 6:**
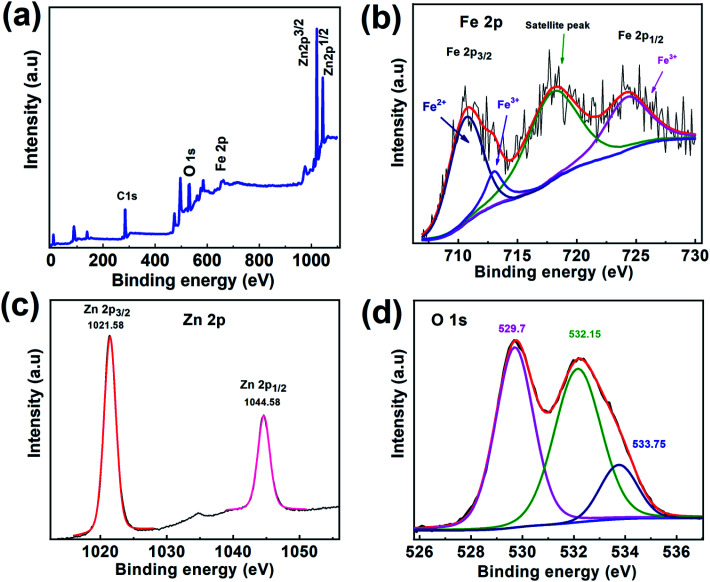
(a) XPS survey spectra and (b) high-resolution XPS spectra of Zn 2p for Fe_3_O_4_/ZnO 1 : 5 samples; (c) high-resolution XPS spectra of Fe 2p and (d) O1s for Fe_3_O_4_/ZnO 1 : 5 sample.

To further study the surface composition of CSFZ, XPS measurement was conducted for Fe_3_O_4_/ZnO 1 : 5 sample and shown in Fig. 6. From XPS survey spectra of 1–5 sample in Fig. 6a, it can be found that the C, O, Fe and Zn elements coexisted in the sample and the intensity of peak represents for the ZnO phase (Zn 2p) is much larger than that for the Fe_3_O_4_ phase (Fe 2p), revealing the formation of the Fe_3_O_4_/ZnO core–shell nanostructure with the elemental signals from the ZnO shell are much stronger than those from the deeper core of Fe_3_O_4_ in the sample. The high-resolution Fe 2p spectrum (Fig. 6b) shows main peak at 710.64 eV which could be attributed to Fe 2p3/2 of Fe^2+^ spcies, two other main peaks at 724.2 eV and 713.08 eV were correspond to Fe 2p1/2 and Fe 2p3/2 of Fe^3+^ species.^37^ There is a satellite peak observed at 717.79 eV, confirming the presence of high crystalline Fe_3_O_4_ in the samples.^38^ As shown in the high resolution Zn 2p spectrum (Fig. 6c), two main peak of ZnO centered at 1021.58 eV and 1044.58 eV corresponding to Zn 2p3/2 and Zn 2p1/2, suggesting the existence of ZnO.^39^ Interestingly, a strong satellite structure was observed in both of Fe 2p and Zn 2p. The reason for the increasing intensity of satellite peaks is most likely due to the defects found in core–shell systems resulting from the interaction between Fe_3_O_4_ core and ZnO shell. The incorporation of Fe ion or Zn ion at interfaces creates a greater defect density would increase conductivity within the particle, which in turn, could boosting shake-up processes that lead to a strong satellite structure,^34^ confirming the hybridization between Fe_3_O_4_ and ZnO phase in the heterostructures. Additionally, O1s spectrum (Fig. 6d) can be fitted by the Gaussian function with the coincidence of 3 peaks distributed at binding energy of 529.7 eV, 532.26 eV, and 533.75 eV which able to assigned respectively, to metal–O bonds (Fe–O bonds, Zn–O bonds); oxygen defects and oxygen adsorbed on the surface of samples including O_2_, hydroxyl (OH^−^) and carbonate (CO_2_^3−^) groups (O^2−^).^40,41^

### Optical and magnetic properties

**Fig. 7:**
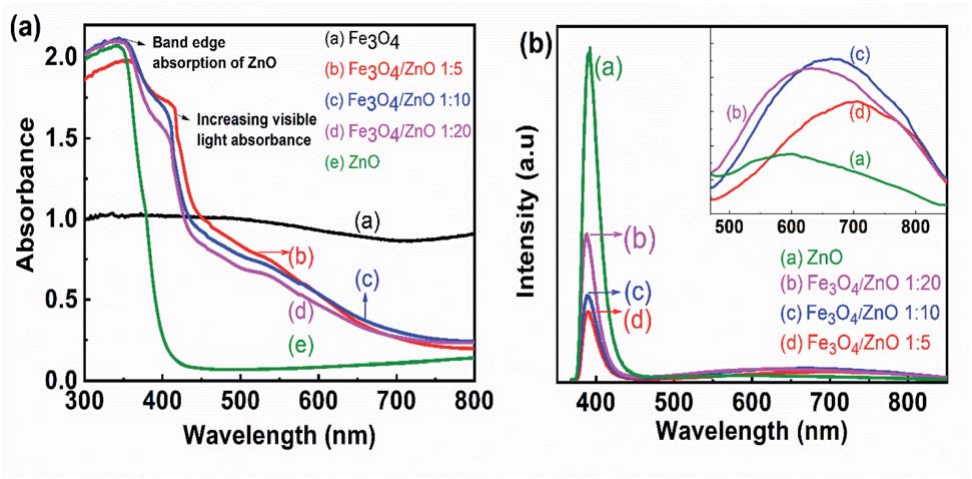
(a) (a) UV-Vis spectra and (b) PL spectra of Fe_3_O_4_ NPs, Fe_3_O_4_/ZnO heterostructures with different Fe : Zn molar ratio: 1 : 5; 1 : 10; 1 : 20.

To determine the band gap of the as-prepared samples, UV-vis absorption spectroscopy was conducted. Fig. 7a shows the UV-vis absorption spectra of prepared samples calculated from the diffuse reflectance by Kubelka–Munk theory.^42^Fig. 7a shows that the control ZnO NPs absorbed light in the UV range; the wavelength corresponding to one set of the absorption edge around 386 nm was consistent with the literature.^43,44^ For the core–shell heterostructures, the absorption spectra showed a shoulder in the region from 400 nm to 450 nm, resulting in double absorption edges. The first absorption edge corresponded to the ZnO absorption edge and another was related to the presence of magnetic NPs due to the forming of core–shell structure with a red shift absorption edges. The appearance of two absorption edges was also observed in previous studies on the composites of semiconductor (ZnO and TiO_2_) and narrow bandgap materials (α-Fe_2_O_3_ and graphene).^45,46^ As shown in Fig. 7a, the second absorption edges shifted toward a longer wavelength with increasing Fe_3_O_4_ ratio. The formation of the core/shell structure with Fe_3_O_4_ as the core and ZnO as the shell offered to couple between two oxides, thereby altering the electronic band structure of ZnO. Combined with these results and the obtained results from FTIR studies, we suggested that the hybridization of Fe_3_O_4_ and ZnO at the core–shell interface created a buffer region in which Fe^3+^ ions were incorporated into the ZnO lattice. The appearance of a shoulder in the absorption spectra of heterostructure samples may be evidence for the existence of the Fe ion-doped ZnO crystal shell. Despite evidence of Fe ions incorporated into the ZnO shell in composite samples from FTIR and UV-vis spectra, the shift in the ZnO diffraction peak was not observed in XRD patterns. Therefore, the core–shell hybridization of Fe_3_O_4_ NPs and ZnO shell formed a Fe ion-doped buffer region at the core–shell interface. Similar results were also found in Fe_3_O_4_/TiO_2_ core–shell NPs as revealed in Stefan's report.^47^ The formation of an impurity level near the bottom of the conduction band CB or the top of the valance band by doping anions or cations enables the second absorption of visible light in core–shell heterostructure samples.^44,46–48^ In addition, a strong UV emission band assigned to the near band edge emission in ZnO was observed at 390 nm in the photoluminescence spectra of composite samples (as shown in Fig. 7b). Another broad visible emission (from 500 nm to 900 nm), which was related to impurity energy in the ZnO crystal, shifted to long wavelengths (inset of Fig. 7b) as the Fe_3_O_4_ core ratio increased. These obtained results may lead to an assumption that the optical properties of the heterostructure samples were simultaneously contributed by pure ZnO material, the buffer region, and Fe_3_O_4_ NPs. The interaction of ZnO and Fe_3_O_4_ NPs at the core–shell interface resulted in the Fe-doped ZnO buffer region. The formation of Fe s-levels below the conduction band edge of ZnO effectively extended the absorption edge into the visible region.^46^ To understand the results better, this feature should be studied further in future work.

**Fig. 8:**
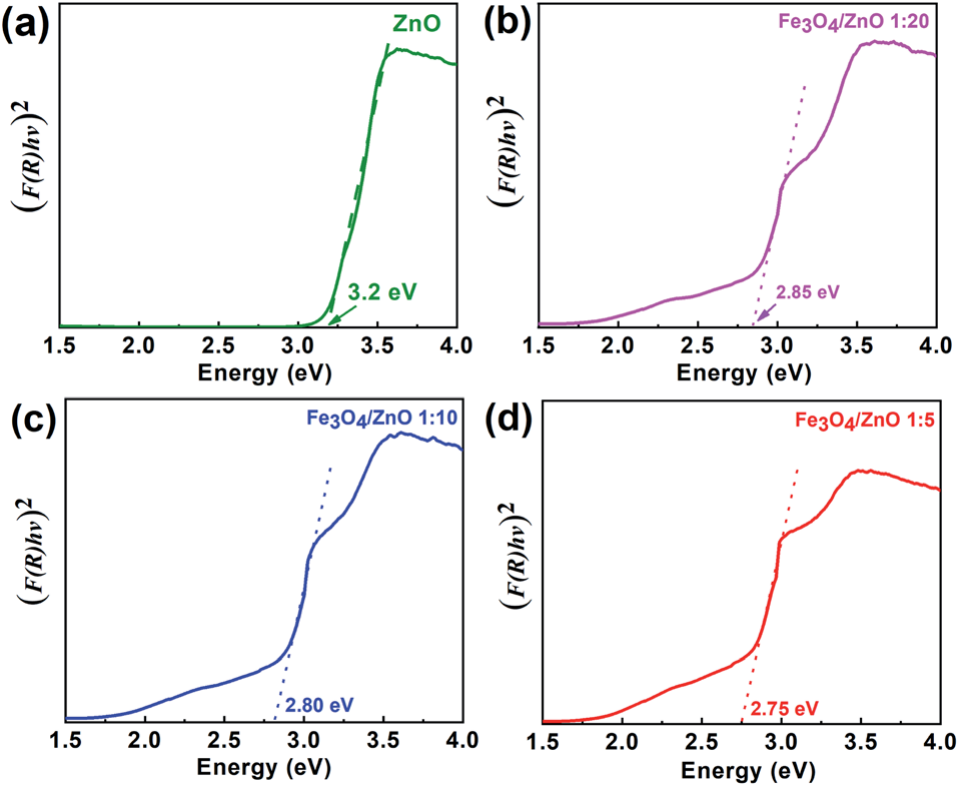
The plot of transformed Kubelka–Munk function *versus* the energy of light for the calculation of *E*_g_, (a), (b), (c) and (d) for as-prepared samples ZnO, Fe_3_O_4_/ZnO 1 : 5; Fe_3_O_4_/ZnO 1 : 10; Fe_3_O_4_/ZnO 1 : 20, respectively.

**Fig. 9:**
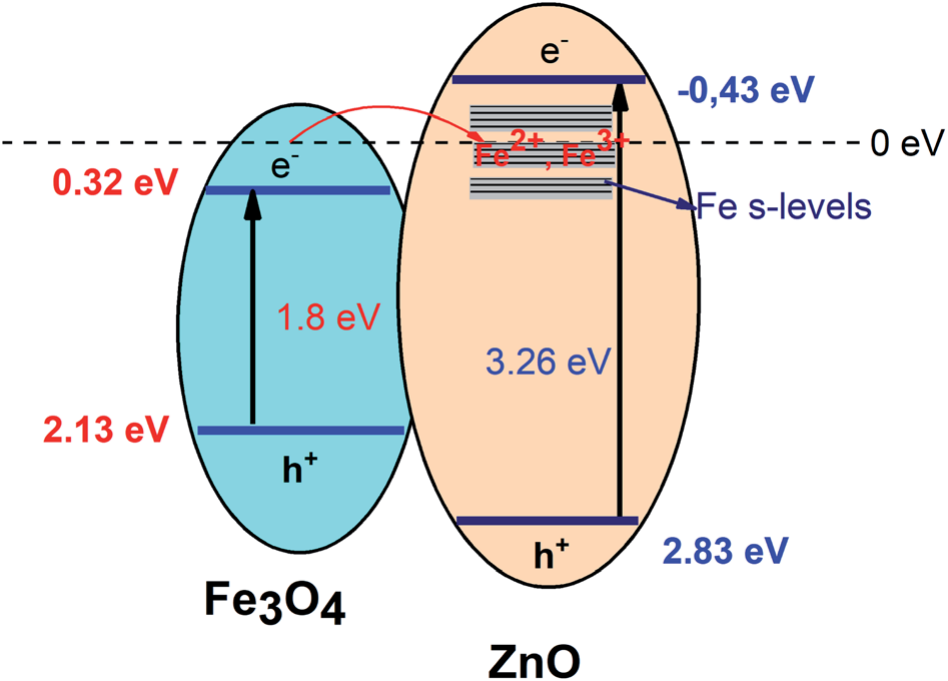
The possible charge transfer mechanism in the Fe_3_O_4_/ZnO core–shell heterostructures.

For further understanding optical properties of the core–shell heterostructures, bandgap values and the possible charge transfer mechanism were explored. The band gap (*E*_g_) was estimated by employing the Kubelka–Munk equation.^49^Fig. 8 show the curves of (*F*(*R*∞)*hv*)^2^*versus* (*hv*) of ZnO NPs and core/shell Fe_3_O_4_/ZnO 1 : 5, 1 : 10 and 1 : 20. The band gap of samples was obtained by extrapolating the linear portion of the (*F*(*R*∞)*hν*)^2^*versus* (*hν*) curve to *F*(*R*∞) = 0. The bandgap of Fe_3_O_4_/ZnO 1 : 5 is 2.75 eV, which is lower than those of Fe_3_O_4_/ZnO-1 : 20 (2.85 eV) Fe_3_O_4_/ZnO 1 : 10 samples (2.81 eV), ZnO NPs (3.26 eV) and Fe_3_O_4_ (1.81 eV). The decreasing in band gap energy of CSFZ with increasing Fe_3_O_4_ core ratio indicated that the visible light adsorption greatly enhanced, which is benefit for the high-efficiency in visible light-driven photocatalytic performance.^46^ This is explained that the unique small core/shell heterostructure and their coupling between Fe_3_O_4_ and ZnO. Based on the calculated bandgap values of Fe_3_O_4_ and ZnO samples, the band edge positions of the valence band (*E*_VB_) and conduction band (*E*_CB_) potentials of Fe_3_O_4_ core nanoparticles and ZnO shell were estimated by the empirical formula:^50^2
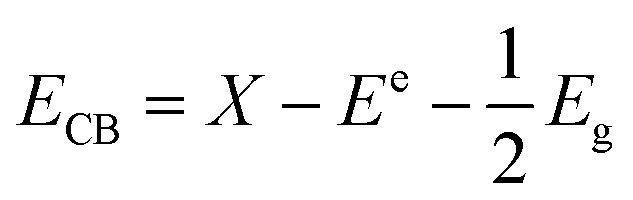
3
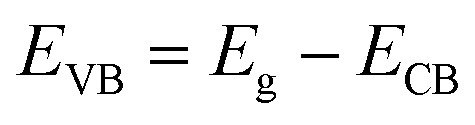
where, *X* is Mulliken's absolute electronegativity (5.73 eV for Fe_3_O_4_ and 5.78 for ZnO eV), *E*^e^ is the energy of free electrons on hydrogen scale (4.5 eV),^51^*E*_g_ is band gap values. *E*_CB_, *E*_VB_ of Fe_3_O_4_ core and ZnO shell were obtained as 0.32 eV, 2.13 eV and −0.43 eV and 2.83 eV, respectively. The possible charge transfer mechanism of the Fe_3_O_4_/ZnO core–shell nanoparticles were insulated in Fig. 9. The charge transfer goes through the interface from Fe_3_O_4_ to ZnO shell and filed Fe s-levels with the incorporated of Fe^2+^, Fe^3+^ ions which may lead to extend the bottom of ZnO conduction band.

### Photocatalytic performance

**Fig. 10:**
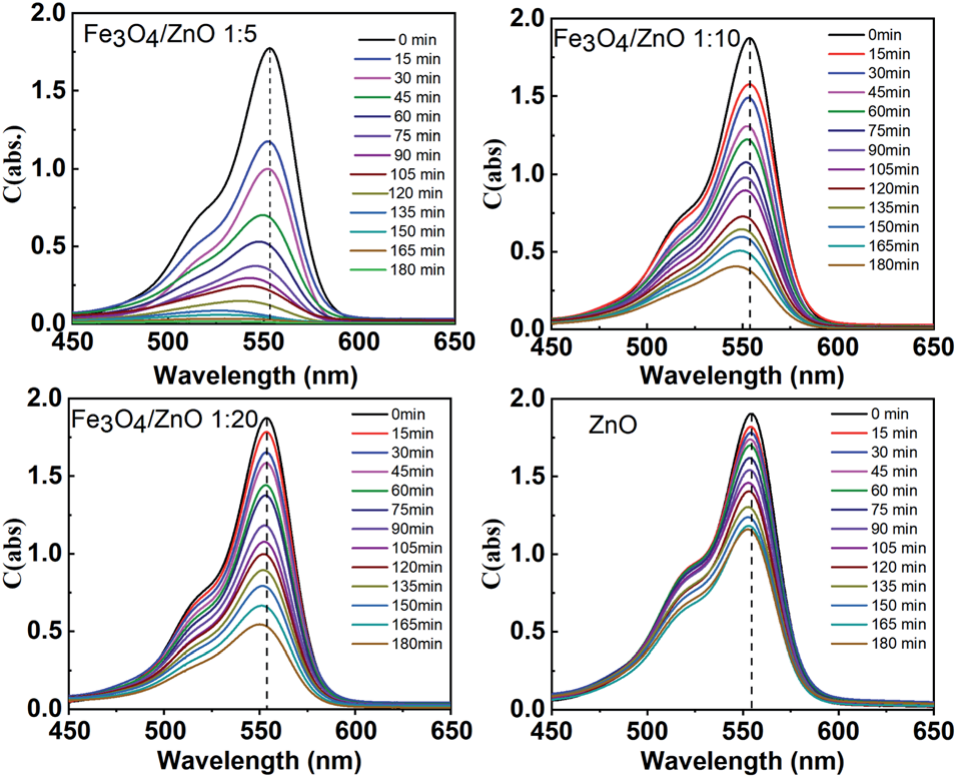
Absorption spectra of the RhB solution as a function of sunlight irradiation time in the presence of Fe_3_O_4_/ZnO heterostructures with different Fe : Zn molar ratio (1 : 5; 1 : 10 and 1 : 20).

**Fig. 11:**
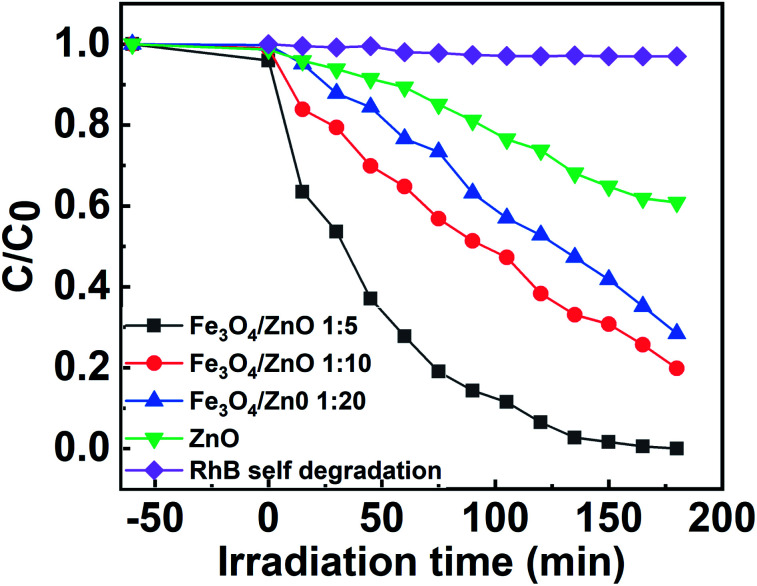
The efficiency of photocatalysts and the RhB self-degradation against the reaction time.

The photocatalytic activity of all the core–shell photocatalysts was examined by monitoring degradation kinetic of aqueous RhB solution under simulated sunlight irradiation. Typically, the experiments were conducted in dark for 1 h to achieve the adsorption equilibrium. Fig. 10 shows the absorption spectra of RhB after photodegradation under the presence of photocatalysts with different irradiation time intervals. The efficiency of photocatalysts and the RhB self-degradation against the reaction time are shown in Fig. 11. Indeed, the photodegradation of Fe_3_O_4_/ZnO 1 : 5 samples was fast and outperformed over the Fe_3_O_4_/ZnO 1 : 10, Fe_3_O_4_/ZnO 1 : 20 samples and ZnO NPs. The Fe_3_O_4_/ZnO 1 : 5 exhibited the highest RhB removal efficiency at 150 min under the sunlight irradiation. Moreover, without photocatalyst, RhB self-degradation under solar light irradiation can be neglected. The enhanced photocatalytic properties in core–shell heterostructures can be governed by two main physicochemical features: (i) low recombination rate of photogenerated charges compared with the charge transfer rate to the surface-bound redox reactions; and (ii) low bandgap energy is provided the material with the ability to absorb a wide range of light in the visible region. As shown in Fig. 11, after reaching the adsorption/desorption equilibrium, the adsorption capacity toward the organic pollutant of all samples was not significantly different. This result implied that the enhanced photocatalytic performance of the core–shell Fe_3_O_4_/ZnO samples was affected by the remaining two factors. The recombination behaviors of the photogenerated electron–hole pairs in the core–shell Fe_3_O_4_/ZnO samples were characterized by PL spectra. As shown in Fig. 7b, the PL emission intensity of core/shell samples significantly decreased with increasing Fe_3_O_4_ core ratio, the weakest PL emission was found in Fe_3_O_4_/ZnO 1 : 5 sample. These results reveal that Fe_3_O_4_/ZnO 1 : 5 sample is more effective in improving charge carrier transfer and separation than those of Fe_3_O_4_/ZnO 1 : 10 and 1 : 20 because the PL emission intensity is proportional to the photo-induced electron–hole recombination rate.^50,52^ It can be explained that the recombination of photogenerated charge carriers in core–shell samples was effectively suppressed by coupling of Fe_3_O_4_ core and ZnO shell with the presence of the impurities of Fe ions incorporated to ZnO lattice at the interface region. According to the work done by Liu *et al.*, the incorporation of Pt^2+^ ions into α-Fe_2_O_3_ photocatalyst induced the formation of Schottky barrier at the interface between two phase of the nanocomposites that can effectively improve charge carrier separation and hinder carrier recombination.^50^ As mentioned in the FTIR results, the addition of Fe ions into the ZnO lattice in Fe_3_O_4_/ZnO 1 : 5 sample is larger than that in Fe_3_O_4_/ZnO 1 : 10 and 1 : 20 samples, which produce more capturing sites to facilitate the separation efficiency of photogenerated carriers. The energy level of the conduction band of ZnO was higher than the Fermi level of Fe_3_O_4_, and photogenerated electrons in the conduction band of ZnO were quickly transferred from ZnO shells to the core.^53^ Thus, the buffer region acted as a reservoir for the photoelectrons and extended the lifetime of the photogenerated charges due to the reduction of Fe^+3^ to Fe^+2^.^21,54^ Thus, the enhanced photocatalytic activity can be achieved in the core–shell Fe_3_O_4_/ZnO nanocomposite, especially in the sample with a core–shell ratio of 1 : 5.

It is well known that the photocatalytic process to degrade organic pollutants involved three reactions: (i) generating electrons and hole at conduction band and valence band of photocatalyst by light absorption; (ii) electrons and hole move to the surface of photocatalyst and take reaction with O_2_ and H_2_O absorbed on photocatalyst's surface to create reactive species; (iii) reactive species will degrade organic pollutants through strong oxidation and reduction reaction.

As shown in Fig. 9, it can be seen that the potential of conduction band of ZnO is more negative than the O_2_/O_2_%^−^ potential (−0.33 eV *vs.* NHE). Thus, photogenerated electrons can react with O_2_ in the solution to generate O^2−^. Additionally, photoinduced h^+^ also can react with H_2_O or hydroxyl group on the surface of photocatalyst to produce ˙OH because the position of valance band potential for ZnO is larger than the standard redox potential of OH (2.7 eV *vs.* NHE). According to recently reports by Han *et al.*, O_2_^−^. ˙OH and h^+^ were found as the main oxidizing species for the photocatalytic process of the ZnO-based composites.^55,56^

### Magnetic properties and reusability of the catalyst

**Fig. 12:**
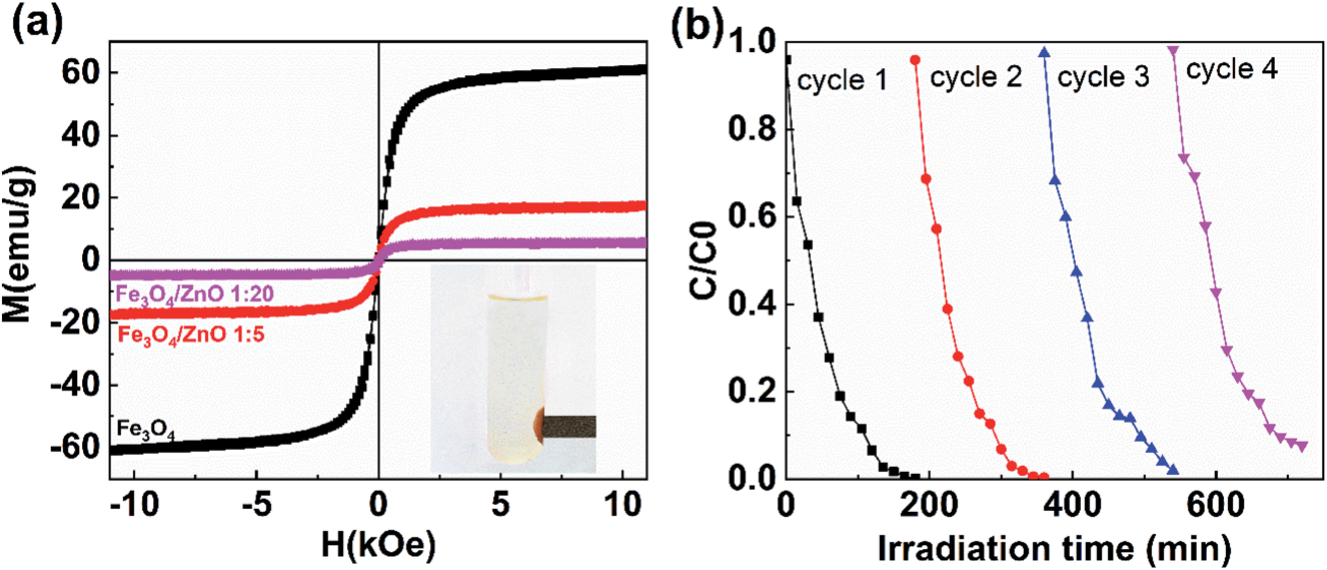
(a) The magnetization curves of uncoated Fe_3_O_4_ nanoparticles and Fe_3_O_4_/ZnO heterostructures with different core–shell ratio of 1 : 5 and 1 : 20; (b) four cycles of photocatalytic degradation of RhB in the present of Fe_3_O_4_/ZnO heterostructures with core–shell ratio of 1 : 5.

The separation and recyclability of a magnetic-based photocatalyst depend strongly on its magnetic properties. Fig. 12a shows the magnetization curves of Fe_3_O_4_ NPs and core/shell Fe_3_O_4_/ZnO (1 : 5 and 1 : 20) measured at 300 K. The saturation magnetization values for Fe_3_O_4_ NPs, Fe_3_O_4_/ZnO 1 : 5, and Fe_3_O_4_/ZnO 1 : 20 were 61, 17.4, and 5.2 emu g^−1^, respectively, which is still comparable to the reported values in the ref. 16, 19 and 53. The reduction of saturation magnetization in core/shell samples was due to the non-magnetic phase of ZnO shell. Interestingly, Fe_3_O_4_/ZnO 1 : 5 could be effortlessly separated and detached from solutions containing RhB by applying an external magnetic field (inset in Fig. 12a). Reusability is an essential feature of composites for organic pollutant photodegradation in practice. The recycling and sustainability of Fe_3_O_4_/ZnO 1 : 5 were further evaluated with four continuous cycles (Fig. 12b). For each cycle, the photocatalyst in solution was rapidly separated by a magnet with surface magnetic field strength of 4 kOe. The solar light photocatalytic activities of sample remained good after four recycles. These results showed that core/shell Fe_3_O_4_/ZnO heteronanostructure could be an excellent candidate for practical wastewater treatment.

## Conclusion

Magnetically separable core–shell Fe_3_O_4_/ZnO heteronanostructures for enhanced solar photocatalytic activity were successfully fabricated by ethylene glycol–citric acid assisted the effective sol–gel process. By controlling the core–shell molar ratio during synthesis, the structure–morphology of the Fe_3_O_4_/ZnO heterostructures varied from nearly spherical to rice seed-like. The formation of the core–shell structure and the hybridization of Fe_3_O_4_ and ZnO at the core–shell interface created a buffer region, in which Fe ions were incorporated into the ZnO lattice. Compared with pristine ZnO, the band gap of the core–shell heterostructures could be significantly modified which enhancing the sunlight harvesting ability. The minimum band gap energy was 2.783 eV, which belonged to the spherical nanocomposite with the core–shell ratio of 1 : 5. In comparison with ZnO NPs, the core–shell nanocomposites showed the higher sunlight photocatalytic activity. Efficient sunlight harvesting and reduced photogenerated electron–hole recombination rate should be the main factors to enhance the sunlight photocatalytic performance of Fe_3_O_4_/ZnO nanocomposites. Our results revealed that core–shell Fe_3_O_4_/ZnO heterostructure with Fe : Zn molar ratio is 1 : 5 showed an excellent sunlight photocatalytic performance, efficient magnetic separation, and recyclability during four cycles. Therefore, this photocatalysts can be excellent candidate for visible-light-driven photocatalysis applications.

## Conflicts of interest

There are no conflicts to declare.

## Supplementary Material
